# *Alternanthera sessilis* Red Ethyl Acetate Fraction Exhibits Antidiabetic Potential on Obese Type 2 Diabetic Rats

**DOI:** 10.1155/2013/845172

**Published:** 2013-03-28

**Authors:** Kok Keong Tan, Kah Hwi Kim

**Affiliations:** Department of Physiology, Faculty of Medicine, University of Malaya, 50603 Kuala Lumpur, Malaysia

## Abstract

The antidiabetic potential of *Alternanthera sessilis* Red was investigated using the obese type 2 diabetic rats induced by high fat diet and streptozotocin. Three fractions (hexane, ethyl acetate, and water) were obtained from the crude ethanol extract of *Alternanthera sessilis* Red. *Alternanthera sessilis* Red ethyl acetate fraction (ASEAF) was found to possess the most potent antihyperglycemic effect through oral glucose tolerance test. The ASEAF was subsequently given to the diabetic rats for two weeks. It was found that two-week administration of ASEAF reduces the fasting blood glucose level, triglyceride level, and free fatty acid level of the rats. ASEAF-treated diabetic rats showed higher pancreatic insulin content and pancreatic total superoxide dismutase activity compared to the untreated diabetic rats. Also, the insulin sensitivity indexes suggested that ASEAF ameliorates the insulin resistant state of the diabetic rats. In conclusion, ASEAF could be developed into a potential antidiabetic agent for the management of type 2 diabetes.

## 1. Introduction

Type 2 diabetes mellitus (T2D) is a group of metabolic disorders that affect more than 90% of the diabetes population. Prevalence of diabetes mellitus in Malaysia is increasing alarmingly. As mentioned by Shaw et al., the national prevalence of diabetes in Malaysia in year 2011 is 11.7% [1]. This is contributed by several factors such as diabetes care facilities, ethnicity, socioeconomic status, urbanization, and most importantly the western lifestyles adopted [2]. With the undesirable side effects of the antidiabetic agents found in the market nowadays, traditional medicine serves an important role in the discovery of new antidiabetic agent with lesser and fewer side effects. One classic example is the alkaloid berberine. Berberine is an inhibitor of dipeptidyl peptidase IV [3]. Yin et al. [4] showed that berberine has similar antihyperglycemic potency to metformin (a common antidiabetic agent) and possessed antihypertriglyceridemic effect which is not seen in metformin. Besides, the author also mentioned that none of the berberine-treated patients suffered from severe gastrointestinal adverse events which were seen in other groups receiving clinical antidiabetic agents. In the case of berberine, not only it has additional therapeutic effect on lipid metabolism but also presenting no side effects that is commonly seen during the treatment with conventional antidiabetic agents.

The wild type of *A. sessilis* has green aerial parts. A decoction of *A. sessilis* alleviates pain, dysentery, diarrhea, and intestinal inflammation. Also, *A. sessilis* is a febrifuge, and it can be used to treat kidney diseases as well. It is often consumed as vegetable in India. Several therapeutic benefits of the wild (green) *A. sessilis* had been investigated which include anti-inflammatory effect [5], the nootropic activity [6], cytotoxic effect towards pancreatic cancer cell lines [7], and the free radical-scavenging ability [8]. The cultivar of this plant has red instead of green aerial parts. This cultivar is more commonly found in Malaysia and Singapore than the wild type. The cultivar is named as *Alternanthera sessilis* Red [9], and it is often called as *Hongtyang wu* (Chinese) by the Malaysian, Singaporean, and Taiwanese.

Traditionally, this cultivar is believed to be able to reduce the risk of cardiovascular disease. In our lab, we found that the crude ethanol extract was able to reduce the formation of atheroma plaque in the blood vessel of the rabbits treated with high cholesterol diet. On the other hand, the crude ethanol extract showed blood glucose lowering effect in the preliminary study carried out in our lab. In the present study, we further explored the antidiabetic potential of the crude ethanol extract and identified the active fraction which is responsible for the antidiabetic effect as well as the physiological mechanism of antidiabetic action.

## 2. Methods

### 2.1. Preparation of Crude Ethanol Extract and Fractions from **Alternanthera sessilis ** Red

The plant *Alternanthera sessilis* Red (ASR) were obtained from Bukit Tinggi, Selangor, Malaysia. It was identified by a botanist Dr. Sugumaran Manickam, and a voucher specimen was deposited in the herbarium at Rimba Ilmu of University of Malaya (KLU 47693). 

The aerial parts of ASR were dried in the oven at 40°C and crushed into powder. One kilogram of the powder was extracted with 3 L of 95% ethanol at room temperature for three days. This extraction step was repeated three times. The mixture was then filtered with Whatman number 1 filter paper. The solvent was removed using RV10 rotary evaporator (IKA, Guangzhou), and the resulting residue was the crude ethanolic extract (112.5 g).

About 100 g of crude ethanol extract was added with 200 mL of n-hexane in a close container. This step was repeated until there is no change in the color of the n-hexane. The mixture was filtered, and the filtrate was concentrated by the rotary evaporator, yielding the hexane fraction (ASHXF). The hexane-insoluble residue was subjected to partitioning between ethyl acetate and distilled water in a separatory funnel. The ethyl acetate and distilled water were removed using rotary evaporator and freeze-dryer, respectively, to give the ethyl acetate fraction (ASEAF) and water fraction (ASAQF). The amount of ASHXF, ASEAF, and ASAQF is 42.1 g, 39.7 g, and 18.2 g, respectively.

### 2.2. Induction of Obese Type 2 Diabetes in Rats

Male Sprague-Dawley (SD) rats weighed between 200–230 g were obtained from the Laboratory Animal Centre, Faculty of Medicine, University of Malaya. The rats were housed in the animal room in Department of Physiology, Faculty of Medicine, University of Malaya, under standard environmental conditions, relative humidity, and dark/light cycle. All animals were acclimatized for one week before any experimental procedures, and all experimental procedures were approved by the animal ethical committee of University of Malaya (Ethic number: FIS/27/01/2010/TKK (R)).

The induction of diabetes was done according to Srinivasan et al. [10] with slight modification. Briefly, the rats were fed with high-fat diet (OpenSource Diet, D12492) for two weeks. On day 15, the overnight fasted (16 hours) rats were injected with 40 mg/kg of streptozotocin (STZ) intraperitoneally. After the injection of STZ, the rats were given 5% glucose solution for 24 hours to prevent hypoglycemia. Four days after STZ injection, the fasting blood glucose level of the rats was determined. Rats with blood glucose level more than 16.5 mmol/L were selected for subsequent experiment. 

### 2.3. Determination of Active Fraction

The blood glucose lowering effect of the fractions was determined by oral glucose tolerance test (OGTT) [11]. Briefly, the diabetic rats were divided into 5 groups (*n* = 5) and were fasted overnight for 16 hours. On the next day, the diabetic rats in different groups were fed with 500 mg/kg fractions (hexane, ethyl acetate, and water), 30 mg/kg glibenclamide (positive control), and a dose volume of 2 mL/kg of 1% carboxymethylcellulose (CMC) prepared in distilled water (negative control), respectively. All fractions and glibenclamide were dissolved in 1% carboxymethylcellulose. After 30 minutes, the blood glucose level of the diabetic rats was measured using a glucometer (AccuChek Advantage). Subsequently, 2 g/kg glucose solution (dissolved in distilled water) was administered to all the diabetic rats. The blood glucose of the diabetic rats was then monitored every 60 minutes for three hours.

An OGTT curve was plotted, and the area under the curve was calculated using the trapezoid rule [12] as follows:

(1) $$\begin{array}{c} \text{AUC}=\frac{C1+C2}{2}\times \left(t2-t1\right), \end{array}$$

where *C*1 and *C*2 are blood glucose concentrations at time points *t*1 and *t*2, respectively.

### 2.4. Effect of Two-Week Administration of ASEAF on the Diabetic Rats

Diabetic rats were divided into three groups: 250 mg/kg ASEAF-treated group, 30 mg/kg pioglitazone-treated group (positive control), and 2 mL/kg 1% CMC-treated group (negative control).

#### 2.4.1. Sample Collection

Blood samples were collected from the tail vein. The blood glucose levels were determined using a glucometer. The pancreas and liver were harvested after the rats being sacrificed through cervical dislocation.

#### 2.4.2. Biological Assays

Plasma and serum samples were prepared from the blood samples collected according to the instructions provided in the assay kits.

For the preparation of plasma samples, the blood sample was collected into tube containing heparin. Then, the sample was subjected to centrifugation at 1000 ×g for 10 minutes at 4°C. The top layer is the plasma sample.

For the preparation of serum samples, the blood sample collected was first allowed to clot for 30 minutes at room temperature. Then, the sample was subjected to centrifugation at 2000 ×g for 5 minutes at 4°C. The top layer is the serum sample.

The samples were labeled and stored at −80°C. On the day of experiment, the samples were thawed at room temperature. The organs harvested were washed with saline solution and stored at −80°C until used.

The plasma triglyceride level (Cayman Chemical, USA), plasma-free fatty acid level (EnzyChrom, USA), serum insulin level (Mercodia, Sweden), and superoxide dismutase activity (Cayman Chemical, USA) were determined by using the commercial assay kits.

#### 2.4.3. Liver Triglyceride Content

Adopted from Lian et al. [13], 0.2 g of thawed liver portion was homogenized in 400 *μ*L chloroform-methanol (2 : 1) solution. The mixture was subjected to sonication for 15 minute at room temperature. Subsequently, the mixture was centrifuged at 5000 rpm for 10 minutes. The supernatant was washed with 0.2 volume of 0.9% NaCl saline and centrifuged again at 2000 rpm for 5 minutes. Two phases were obtained, and the lower phase was evaporated. The residual was dissolved in 0.5 mL of isopropanol which contained 10% Triton X-100 before subjected to the triglyceride assay using the assay kit. The liver triglyceride content was expressed as mg/g wet tissue.

#### 2.4.4. Pancreatic Insulin Content and Pancreatic Total Superoxide Dismutase Activity

The extraction of pancreatic insulin was adopted from Portha et al. [14]. 0.2 g of thawed pancreas portion was placed in a centrifuge tube containing 5.0 mL of ice-cold acid-alcohol solution. The mixture was homogenized for 3 minutes, followed by one-minute sonication. The solution was left to stand at −20°C overnight and centrifuged at 3000 rpm at 4°C for 15 minutes in the next day. The supernatant was transferred into a new centrifuge tube and stored at −20°C, while the pellet was subjected to extraction again. Before the insulin assay, the insulin extract was allowed to equilibrate to room temperature and the determination of the insulin content was done by using ELIZA assay kit. The pancreatic insulin content was expressed as *μ*g/mg wet tissue.

About 0.2 g of thawed pancreas portion was homogenized in 5.0 mL of cold 20 mM HEPES buffer, pH 7.2. The mixture was subjected to centrifugation at 3000 rpm for 5 minutes at 4°C. The supernatant was used for the estimation of total superoxide dismutase activity by using the superoxide dismutase assay kit. The superoxide dismutase activity was expressed as unit/mg wet tissue.

#### 2.4.5. Insulin Sensitivity Indexes (HOMA and QUICKI)

The homeostasis model assessment (HOMA) and quantitative insulin sensitivity check index (QUICKI) were calculated according to Cacho et al. [15] as follows:

(2) $$\begin{array}{c} \text{HOMA}=\frac{\text{fasting}\;\text{BGL}\times \text{fasting}\;\text{SIL}}{2430},\\ \text{QUICKI}=\frac{1}{\log_{10}\left(\text{fasting}\;\text{BGL}\right)+\log_{10}\left(\text{fasting}\;\text{SIL}\right)}, \end{array}$$

where the fasting BGL was in mg/dL, while the fasting SIL was in *μ*U/mL.

### 2.5. Preliminary Phytochemical Screening

The ASEAF was subjected to a preliminary phytochemical screening. ASEAF was dissolved in dichloromethane and spotted on a thin-layer chromatography (TLC) plate. The TLC plate was then allowed to develop in a chamber saturated with ethyl acetate and chloroform in the ratio of 6 : 4 (ethyl acetate : chloroform). Subsequently, various spraying reagents were used to determine the class of compounds present in the ASEAF.

### 2.6. Statistical Analysis

Values were represented as mean ± standard error from mean (SEM) with “*n*” represents sample size. Paired and unpaired Student's *t*-test was conducted using SPSS 17.0. A *P* value less than 0.05 (*P* < 0.05) was considered significant.

## 3. Results

### 3.1. Determination of Active Fraction

**Figure 1:**
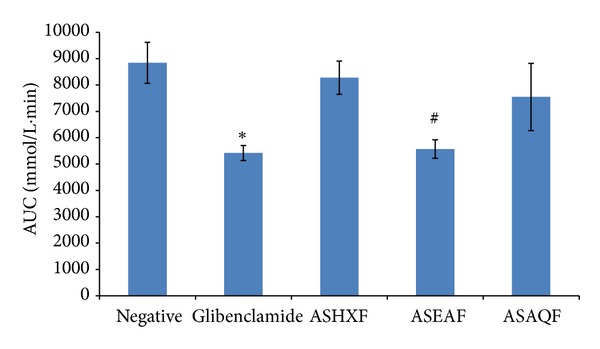
Effect of different fractions of the crude ethanolic extract of *Alternanthera sessilis* Red on OGTT. The diabetic rats were administered with 500 mg/kg of hexane fraction (ASHXF), ethyl acetate fraction (ASEAF), and water fraction (ASAQF). The data shown are the AUC of OGTT and are expressed as mean ± SEM, 5 rats (*n* = 5) per group. *P < 0.05,  ^#^ *P* < 0.01 versus negative control group.

Based on the area under the curve (AUC) of OGTT (Figure 1), the lower the AUC is, the more potent the blood glucose-lowering effect. In this experiment, glibenclamide is a clinical anti-diabetic agent and was used as positive control in the OGTT. It is clear that 10 mg/kg of glibenclamide significantly suppressed the rise in the BGL (*P* < 0.05) when compared with the negative control group which received 1% CMC. Among the fractions, 500 mg/kg EAF produced a more significant blood glucose-lowering effect than glibenclamide (*P* < 0.01) when compared with the negative control group. HXF and AQF did not show significant antihyperglycemic effect in this experiment (*P* > 0.05). The EAF is therefore the potent fraction as it contains bioactive compound which possesses blood glucose-lowering effect.

### 3.2. Determination of Effect of Two-Week Administration of ASEAF on the Diabetic Rats

**Figure 2:**
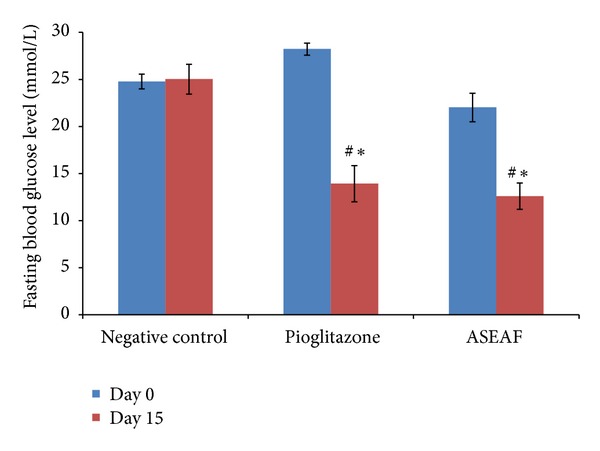
Effect of ASEAF on the fasting blood glucose level of the diabetic rats after two-week administration of 250 mg/kg ASEAF. Data shown are the fasting blood glucose level (mmol/L) and are expressed as mean ± SEM 6 rats (*n* = 6) per group. *P < 0.05 versus negative control; ^#^ *P* < 0.05 versus day 0 value (before treatment).

**Figure 3:**
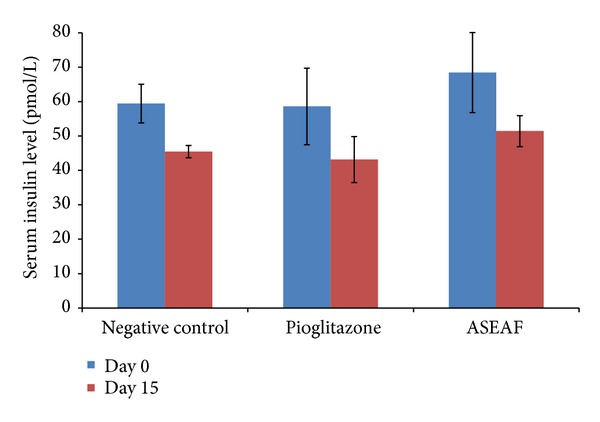
Effect of ASEAF on the fasting serum insulin level of the diabetic rats after two-week administration of 250 mg/kg ASEAF. Data shown are the fasting serum insulin level (pmol/L) and are expressed as mean ± SEM, 6 rats (*n* = 6) per group. No significant difference was found.

In the two-week study, 30 mg/kg pioglitazone (a potent insulin sensitizer) was used as a positive control, whereas the negative control group consisted of diabetic rats given with 2 mL/kg of 1% CMC (the vehicle). The fasting blood glucose level (BGL) of both ASEAF-treated group and pioglitazone-treated group was significantly lower than the negative control group (*P* < 0.01) and their respective day 0 values (*P* < 0.05) as shown in Figure 2. However, the serum insulin levels (SIL) of both groups did not show any significant difference (*P* > 0.05) when compared with the negative control group and their respective day 0 values (Figure 3). 

The salient point is that the decrease in the fasting BGL leads us to reckon that the possible mechanism of action of ASEAF was through the alleviation of insulin resistance. Hence, the HOMA and QUICKI were computed. As shown in Table 1, the HOMA indexes of the ASEAF-treated group and pioglitazone-treated group were significantly lower than the negative control group (*P* < 0.001) and their respective values before treatment (*P* < 0.05 and *P* < 0.001, resp.). On the other hand, the QUICKI values of both treatment groups were significantly higher than the negative control group (*P* < 0.001) and their respective values before treatment. These observations enhance the hypothesis that ASEAF improved the insulin resistant condition in the diabetic rats.

**Figure 4:**
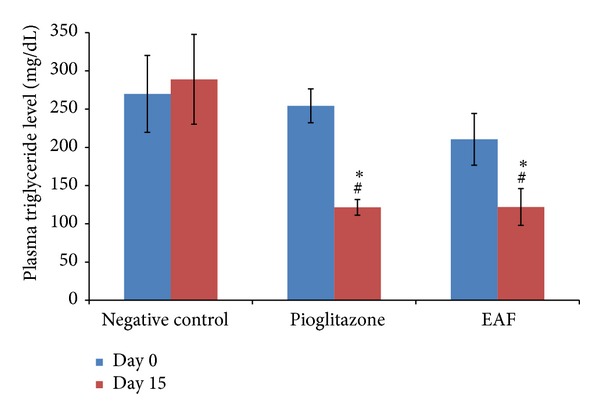
Effect of ASEAF on the fasting plasma triglyceride level of the diabetic rats after two-week administration of 250 mg/kg ASEAF. Data shown are fasting plasma triglyceride level (mg/dL) and are expressed as mean ± SEM, 6 rats (*n* = 6) per group. *P < 0.05 versus negative control; ^#^ *P* < 0.05 versus day 0 value (before treatment).

**Figure 5:**
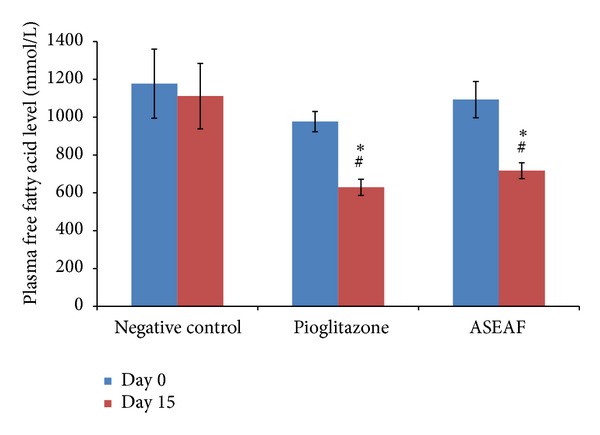
Effect of ASEAF on the fasting plasma-free fatty acid level of the diabetic rats after two-week administration of 250 mg/kg ASEAF. Data shown are fasting plasma free fatty acid level (mmol/L) and are expressed as mean ± SEM, 6 rats (*n* = 6) per group. *P < 0.05 versus negative control; ^#^ *P* < 0.05 versus day 0 value (before treatment).

Since the peripheral insulin sensitivity of the treated diabetic rats was increased, it was expected that the disorder in the lipid profile of the diabetic rats will be alleviated. Indeed, the plasma triglyceride (TG) level and plasma free fatty acid (FFA) level of the pioglitazone-treated group and ASEAF-treated group were significantly lower than the negative control group (*P* < 0.05) as shown in Figures 4 and 5, respectively. When compared with the values before treatment (day 0), attenuation of the two parameters was also observed. After treatment with 30 mg/kg pioglitazone, the plasma TG level, and plasma FFA level of the treated rats decreased 52.21% and 35.57%, respectively. Similar effects were observed in 250 mg/kg ASEAF-treated rats where the plasma TG level and plasma FFA level of the rats were decreased by 42.04% and 34.38%, respectively.

**Figure 6:**
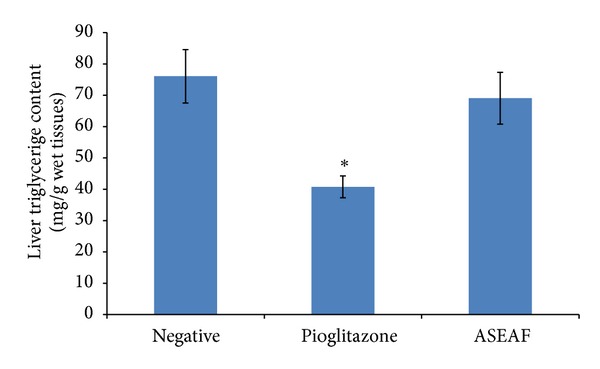
Effect of ASEAF on the liver triglyceride content of the diabetic rats after two-week administration of 250 mg/kg ASEAF. Data shown are liver triglyceride content (mg/g wet tissues) and are expressed as mean ± SEM, 6 rats (*n* = 6) per group. *P < 0.05 versus negative control.

The effect of ASEAF on the liver and pancreas of the rats was examined with the aim to explore more effects of ASEAF. As shown in Figure 6, ASEAF did not significantly alter the liver triglyceride content of the diabetic rats when compared to the negative control group (*P* > 0.05). On the contrary, pioglitazone significantly decreased the liver triglyceride content when compared with the negative control group (*P* < 0.05). 

**Figure 7:**
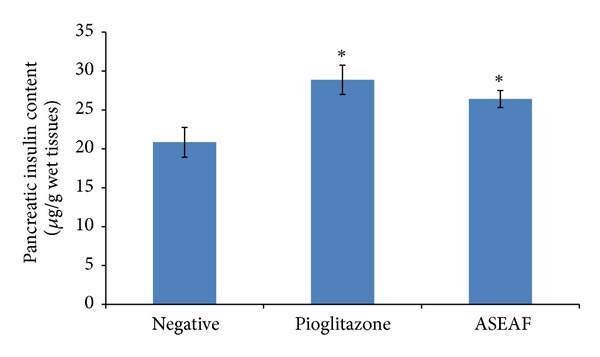
Effect of ASEAF on the pancreatic insulin content of the diabetic rats after two-week administration of 250 mg/kg ASEAF. Data shown are pancreatic insulin content (*μ*g/g wet tissues) and are expressed as mean ± SEM, 6 rats (*n* = 6) per group. *P < 0.05 versus negative control.

**Figure 8:**
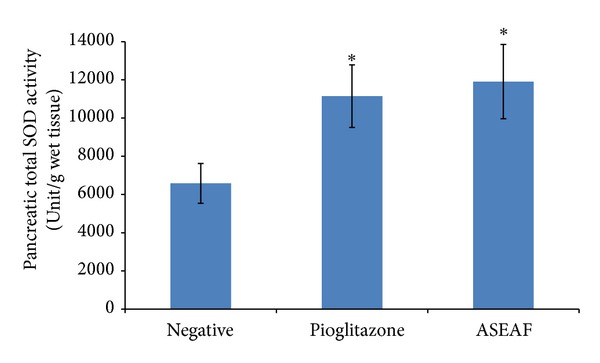
Effect of ASEAF on the pancreatic total superoxide dismutase activity of the diabetic rats after two-week administration of 250 mg/kg ASEAF. Data shown are pancreatic total superoxide dismutase activity (unit/g wet tissues) and are expressed as mean ± SEM, 6 rats (*n* = 6) per group. *P < 0.05 versus negative control.

Considering that the SIL was not significantly altered (*P* > 0.05), it was presumed that the functionality of the pancreas is preserved. As can be seen in Figure 7, both ASEAF and pioglitazone increased the pancreatic insulin content of the diabetic rats (*P* < 0.05). The pancreatic total superoxide dismutase (SOD) activity of the rats was measured to gain insight on the integrity of the antioxidant system of the pancreas.  Figure 8 shows that the pancreatic total SOD activity of both ASEAF-treated group and pioglitazone-treated group was significantly higher than the negative control group (*P* < 0.05).

### 3.3. Phytochemical Screening

Spraying reagents contain chemicals that bind to specific functional group of a compound and producing colors that allows the researcher to identify the presence of a specific class of compound. Preliminary phytochemical screening indicates that ASEAF contains phenolic compound, terpenoids, alkaloid, and secondary amines.

## 4. Discussion

The combination of high fat diet and low dose streptozotocin (STZ) has been commonly used to induce obese type 2 diabetes in the rats [10]. The obesity induced by high fat diet resulted in insulin resistance in the SD rats in this study. The low dose of STZ injected to the insulin resistant rats produced frank hyperglycemia which mimics human type 2 diabetes.

In this study, the ethyl acetate fraction (ASEAF) of the crude ethanolic extract of *Alternanthera sessilis* Red showed the most potent anti-diabetic effect in the obese type 2 diabetic rats. Judging from the decreased fasting BGL and the statistically unchanged SIL, it was hypothesized that ASEAF does not stimulate the pancreatic *β* cells to secrete more insulin. Rather, it improved the peripheral insulin sensitivity. This is because at a similar level of insulin in the blood of the diabetic rats in different groups, the fasting BGL of the treatment groups (ASEAF treated and pioglitazone treated) were lower, indicating that the decrease in BGL was brought about by the enhanced insulin action. Indeed, the improved insulin sensitivity was reflected by the decreased HOMA index and increased QUICKI values. HOMA [16] and QUICKI [17] are often used in replacement to the hyperinsulinemic-euglycemic clamp study due to the high positive correlation between these indexes to the results obtained from the hyperinsulinemic-euglycemic clamp study.

ASEAF reduced the plasma FFA level, halting the worsening of the diabetic condition in the rats. When ASEAF increases peripheral insulin sensitivity, lipolysis is suppressed, and FFA level is reduced. ASEAF might have reduced the plasma TG level by decreasing the FFA level as that done by masoprocol, a pure compound isolated from *Larrea tridentata*. Masoprocol inhibits hormone-sensitive lipase (HSL) via, possibly, the dephosphorylation of HSL by increasing phosphatase activity [18]. Pioglitazone improves peripheral insulin resistance by activating peroxisome proliferator-activated receptor-gamma (PPAR*γ*). Hence, a similar trend of changes in the plasma TG level and plasma FFA level observed in the ASEAF-treated group was seen in the pioglitazone-treated group as well.

Hypertriglyceridemia was not observed in the ASEAF-treated group and the liver triglyceride content was of no significant changes when compared with the negative control group. This observation suggests that the antihypertriglyceridemic action of ASEAF might be on other target site such as the musculature or the adipose tissues instead of the liver. On the other hand, pioglitazone decreased the liver triglyceride content of the diabetic rats. Murase et al. [19] suggested that the antidiabetic effect and ameliorative effect of pioglitazone on lipid metabolism abnormalities are associated with its suppressive effect on TNF*α* production.

The liver and muscles are two common sites of insulin resistance with muscle insulin resistance come after hepatic insulin resistance [20]. Plasma FFA level has been known to cause hepatic insulin resistance [21]. When insulin resistance is developed, a vicious cycle takes place, whereby lipid metabolism will be deranged, and more FFA will be produced. The liver abstracts more FFA when the FFA concentration in the blood increases. Subsequently, the hepatocytes synthesize more TG from the FFA and incorporate the TG into VLDL. Furthermore, lipolysis is not suppressed under insulin resistance, producing and releasing more FFA into the circulation. Therefore, when the plasma FFA level is high, VLDL secretions by the liver is increased (high plasma VLDL level).

Chronic FFA-induced insulin hypersecretion reduces insulin biosynthesis in the pancreatic *β* cells [22]. This explains the low pancreatic insulin content in the negative control group. Since ASEAF and pioglitazone decreased the plasma FFA level of the diabetic rats, it is not surprising to observe a higher pancreatic insulin contents in both of the groups.

The pancreatic total superoxide dismutase activity of both of the treatment groups was higher than the negative control group. Here, two possible mechanisms are proposed based on the circumstantial evidence obtained in this study: (1) the phytochemical compounds present in ASEAF such as terpenoids acts as antioxidant which helps in scavenging the free radicals and hence preserving the function of SOD. Terpenoids are known to possessed antioxidative capacity [23]. Quintans-Júnior et al. demonstrated that (+)-camphene, which is a type of terpenoids, possessed powerful antioxidant effect [24], or (2) ASEAF improves peripheral insulin sensitivity which reduces blood glucose concentration. When hyperglycemia is improved, the rate of protein glycation (SOD in this case) can be slowed down. Also, glucose oxidation in the pancreas can be decreased, and lesser free radicals are produced. Thence, the total SOD activity was preserved. Other possible mechanism which might contribute to the higher pancreatic SOD activity includes the increase in interleukin-1 beta which increases the transcription of SOD gene and so the SOD activity [25]. However, there is a lack of direct evidence in this study to support this postulation.

As the diabetic condition progresses, the pancreatic *β* cells are exhausted. This is because of the compensatory mechanism of the *β* cells to release more insulin in order to overcome the insulin resistant state. Persistent hyperglycemia tends to cause glycation of different proteins *in vivo*. Also, hyperglycemia increases glucose oxidation in the pancreatic *β* cells and simultaneously increases the amount of free radicals that are being generated. Superoxide dismutase (SOD) is an important antioxidant system in the pancreas, the Cu-Zn-SOD subtype in particular. Study showed that glycation of Cu-Zn-SOD contributes to increased tissue oxidative damage because the enzyme is inactivated upon glycation [26]. Thus, preserving the function of SOD would no doubt reduce the oxidative damage to the pancreatic *β* cells and maintain the normal function of the *β* cells in insulin biosynthesis and secretion.

The preliminary phytochemical study revealed that the ASEAF contains phenolic compounds, terpenoids, alkaloids, and secondary amines (Table 2).

## 5. Conclusions

We demonstrated that ASEAF possesses antihyperglycemic effect, antitriglyceridemic effect, and pancreatic protective effect in obese type 2 diabetic rats. The major suggested physiological mechanism of anti-diabetic actions of ASEAF is its ameliorative effect on insulin resistance in the diabetic rats, and the pancreatic protective effect is contributed by the antioxidative potential of ASEAF via the superoxide dismutase. Further studies are on the way to pile up more data and evidence in order to elucidate the molecular mechanism of action using both *in vitro* and *in vivo* methods. Also, isolation and identification of the bioactive compound(s) that responsible for the antidiabetic effect are in progress. Overall, ASEAF could be a potential antidiabetic agent for the management of obese type 2 diabetes. 

## Figures and Tables

**Table 1:** Effect of ASEAF on the insulin resistant state of the diabetic rats. The insulin sensitivity indexes (HOMA and QUICKI) were used to quantify the effect. The index values before and after 250 mg/kg ASEAF oral administration were shown.

	HOMA index		QUICKI	
	Before	After	Before	After
Negative	1.563 ± 0.169	1.197 ± 0.038	0.280 ± 0.003	0.289 ± 0.001
Pioglitazone	1.746 ± 0.348	0.646 ± 0.103^a,b^	0.279 ± 0.005	0.316 ± 0.006^a,d^
ASEAF	1.616 ± 0.302	0.689 ± 0.109^a,c^	0.282 ± 0.006	0.313 ± 0.005^a,b^

Data shown are the calculated index values and are presented as mean ± SEM, 6 rats (*n* = 6) per group; ^a^ *P* < 0.001 versus negative control group; ^b^ *P* < 0.01; ^c^ *P* < 0.05; ^d^ *P* < 0.001 versus before treatment.

**Table 2:** Preliminary results of the phytochemical screening on ASEAF. Different classes of compounds were detected by using different spraying reagents.

Reagents	Type of compound	Observations	Inferences
0.5% ninhydrin in acetone	Amino acid	Yellow spots	Absence of amino acid but presence of secondary amine
10% SbCl_3_ in chloroform	Terpenoid	Green zones with different intensities	Absence of carotenoid; presence of terpenoids
Vanillin in H_2_SO_4_: CH_3_COOH:CH_3_OH	Terpenoid	Purple zone	Presence of terpenoids
Dragendorff's reagent	Alkaloid	Orange zone	Presence of alkaloids
5% FeCl_3_ (aq)	Phenolic compound	Some green zones	Presence of phenolic

## References

[1] Shaw JE, Sicree RA, Zimmet PZ (2010). Global estimates of the prevalence of diabetes for 2010 and 2030. *Diabetes Research and Clinical Practice*.

[2] Ismail IS, Wan Nazaimoon WM, Wan Mohamad WB (2000). Socioedemographic determinants of glycaemic control in young diabetic patients in peninsular Malaysia. *Diabetes Research and Clinical Practice*.

[3] Al-Masri IM, Mohammad MK, Tahaa MO (2009). Inhibition of dipeptidyl peptidase IV (DPP IV) is one of the mechanisms explaining the hypoglycemic effect of berberine. *Journal of Enzyme Inhibition and Medicinal Chemistry*.

[4] Yin J, Xing H, Ye J (2008). Efficacy of berberine in patients with type 2 diabetes mellitus. *Metabolism*.

[5] Subhashini T, Krishnaveni B, Srinivas Reddy C (2010). Anti-inflammatory activity of the leaf extract of *Alternanthera sessilis*. *HYGEIA Journal For Drugs and Medicines*.

[6] Surendra Kumar M, Silpa Rani G, Swaroop Kumar SLVVSNK, Astalakshmi N (2011). Screening of aqueous and ethanolic extracts of aerial parts of *Alternanthera sessilis* Linn. R.br.ex.dc for nootropic activity. *Journal of Pharmaceutical Sciences and Research*.

[7] George S, Bhalerao SV, Lidstone EA (2010). Cytotoxicity screening of Bangladeshi medicinal plant extracts on pancreatic cancer cells. *BMC Complementary and Alternative Medicine*.

[8] Borah A, Yadav RNS, Unni BG (2011). In vitro antioxidant and free radical scavenging activity of *Alternanthera sessilis*. *International Journal of Pharmaceutical Sciences and Research*.

[9] Boo CM, Omar-Hor K, Ou-Yang CL (2006). *1001 Garden Plants in Singapore*.

[10] Srinivasan K, Viswanad B, Asrat L, Kaul CL, Ramarao P (2005). Combination of high-fat diet-fed and low-dose streptozotocin-treated rat: a model for type 2 diabetes and pharmacological screening. *Pharmacological Research*.

[11] Pushparaj P, Tan CH, Tan BKH (2000). Effects of *Averrhoa bilimbi* leaf extract on blood glucose and lipids in streptozotocin-diabetic rats. *Journal of Ethnopharmacology*.

[12] Veerapur VP, Prabhakar KR, Thippeswamy BS, Bansal P, Srinivasan KK, Unnikrishnan MK (2011). Antidiabetic effect of *Ficus racemosa* Linn. Stem bark in high-fat diet and low-dose streptozotocin-induces type 2 diabetic rats: a mechanistic study. *Food Chemistry*.

[13] Lian Z, Li Y, Gao J (2011). A novel AMPK activator, WS070117, improves lipid metabolism discords in hamsters and HepG2 cells. *Lipids in Health and Disease*.

[14] Portha B, Picon L, Rosselin G (1979). Chemical diabetes in the adult rat as the spontaneous evolution of neonatal diabetes. *Diabetologia*.

[15] Cacho J, Sevillano J, de Castro J, Herrera E, Ramos MP (2008). Validation of simple indexes to assess insulin sensitivity during pregnancy in Wistar and Sprague-Dawley rats. *American Journal of Physiology*.

[16] Katz A, Nambi SS, Mather K (2000). Quantitative insulin sensitivity check index: a simple, accurate method for assessing insulin sensitivity in humans. *The Journal of Clinical Endocrinology and Metabolism*.

[17] Gowri MS, Azhar RK, Kraemer FB, Reaven GM, Azhar S (2000). Masoprocol decreases rat lipolytic activity by decreasing the phosphorylation of HSL. *American Journal of Physiology*.

[18] Matthews DR, Hosker JP, Rudenski AS (1985). Homeostasis model assessment: insulin resistance and *β*-cell function from fasting plasma glucose and insulin concentrations in man. *Diabetologia*.

[19] Murase K, Odaka H, Suzuki M, Tayuki N, Ikeda H (1998). Pioglitazone time-dependently reduces tumour necrosis factor-*α* level in muscle and improves metabolic abnormalities in Wistar fatty rats. *Diabetologia*.

[20] Kraegen EW, Clark PW, Jenkins AB, Daley EA, Chisholm DJ, Storlien LH (1991). Development of muscle insulin resistance after liver insulin resistance in high-fat-fed rats. *Diabetes*.

[21] Boden G, She P, Mozzoli M (2005). Free fatty acids produce insulin resistance and activate the proinflammatory nuclear factor-*κ*b pathway in rat liver. *Diabetes*.

[22] Bollheimer LC, Skelly RH, Chester MW, McGarry JD, Rhodes CJ (1998). Chronic exposure to free fatty acid reduces pancreatic *β* cell insulin content by increasing basal insulin secretion that is not compensated for by a corresponding increase in proinsulin biosynthesis translation. *Journal of Clinical Investigation*.

[23] Graßmann J (2005). Terpenoids as plant antioxidants. *Vitamins and Hormones*.

[24] Quintans-Júnior L, Moreira JCF, Pasquali MAB (2013). Antinociceptive activity and redox profile of the monoterpenes (+)-camphene, *p*-cymene and geranyl acetate in experimental models. *ISRN Toxicology*.

[25] Borg LAH, Cagliero E, Sandler S, Welsh N, Eizirik DL (1992). Interleukin-1*β* increases the activity of superoxide dismutase in rat pancreatic islets. *Endocrinology*.

[26] Hunt JV, Smith CCT, Wolff SP (1990). Autoxidative glycosylation and possible involvement of peroxides and free radicals in LDL modification by glucose. *Diabetes*.

